# Cornulin as a Potential Novel Biomarker for Cutaneous Squamous Cell Carcinoma

**DOI:** 10.7759/cureus.31694

**Published:** 2022-11-20

**Authors:** Rachna Karumuri, Dean Shah, Hilal Arnouk

**Affiliations:** 1 Osteopathic Medicine, Midwestern University Chicago College of Osteopathic Medicine, Downers Grove, USA; 2 Public Health, Midwestern University Public Health Program, Glendale, USA; 3 Pathology, Midwestern University Chicago College of Osteopathic Medicine, Downers Grove, Downers Grove, USA; 4 Pathology, Midwestern University College of Graduate Studies, Downers Grove, USA; 5 Pathology, Midwestern University College of Dental Medicine, Downers Grove, USA; 6 Pathology, Midwestern University Chicago College of Optometry, Downers Grove, USA; 7 Molecular Pathology, Midwestern University Precision Medicine Program, Downers Grove, USA

**Keywords:** computer-assisted image analysis, immunohistochemistry (ihc), nonmelanoma skin cancer, cutaneous squamous cell carcinoma (scc), cornulin

## Abstract

Background

This study aimed to evaluate the expression of an epidermal differentiation marker, cornulin, in cutaneous squamous cell carcinoma (cSCC). Cornulin has been found to be downregulated in various squamous cell carcinomas of other tissues; however, its expression in cSCC has never been studied. We predicted that cornulin expression in cSCC is reduced compared to the normal epidermis. Moreover, we hypothesized that an inverse relationship exists between cornulin expression and the loss of differentiation, as defined by histopathological grading of cSCC lesions.

Methodology

Samples of normal skin and cSCC lesions of variable histopathological grades were stained using immunohistochemistry. High-resolution tissue images were analyzed with Aperio ImageScope (Leica Biosystems) utilizing a positive-pixel-counting algorithm to quantify the staining intensity. Histo-score (H-score) was calculated based on staining intensity and percentage of positive cell staining. Mean H-scores were compared using an unpaired t-test.

Results

We documented cornulin expression in cSCC for the first time. Cornulin levels were downregulated by more than two-fold in cSCC compared to the normal epidermis. Additionally, we observed a 4.5-fold downregulation in cornulin expression in tumors with high histopathological grades when compared to low histopathological grade tumors.

Conclusions

Cornulin expression levels measured through immunohistochemistry staining can help distinguish among the different histopathological grades of cSCC. Therefore, we propose that cornulin detection can be an adjunct to pathological examination to evaluate the differentiation status of cSCC specimens. Longitudinal studies are needed to establish the utility of cornulin as a diagnostic and prognostic biomarker for cSCC.

## Introduction

Cutaneous squamous cell carcinoma (cSCC) is one of the three most common malignant neoplasms of the skin. It is the second most common cancer of the skin after basal cell carcinoma [1]. The incidence rate of metastatic cSCC is 4%, but in patients with high-risk factors, the metastasis rate is 37% [2-4]. These risk factors include immunocompromised status; large tumor size; localization to the ear, genitals, or lip; perineural or lymphovascular involvement; increased depth of invasion; and high histopathological grades reflecting poor differentiation [5]. This is concerning because the mortality rate of metastatic cSCC is about 70% [6-8]. Moreover, the recurrence rate for metastatic cSCC ranges between 15% and 28% [9,10]. Therefore, cSCC is responsible for the most deaths caused by non-melanoma skin cancer. Additionally, the incidence of cSCC is rising due to increased exposure to ultraviolet radiation and skin damage due to sunbathing, tanning beds, occupational exposure, and decreased ozone from the effects of climate change [11-13]. This may be further exacerbated by the lack of usage of sun protection factor (SPF) sunscreen throughout much of the 20th century. Treatment of cSCC includes surgical resection or topical medications, but a complete recovery is not always attainable, especially in metastatic squamous cell carcinoma (SCC) of the skin. Current diagnostic methods of cSCC primarily rely on histopathologic examination based on morphological features alone. Currently, there are no established diagnostic and prognostic biomarkers for cSCC.

In this study, we investigated the diagnostic and prognostic utility of a novel biomarker, cornulin, an epidermal differentiation marker found in the upper layers of stratified squamous epithelia. Cornulin, encoded by the *CRNN *gene located on chromosome 1q21 locus, is a 495 amino acid protein that belongs to the family of S100 fused-type proteins and is postulated to possess tumor suppressor characteristics [14]. Moreover, cornulin has previously been found to be downregulated in various SCCs of other tissues, such as cervical SCC [15], oral SCC [16], and esophageal SCC [17]. However, no studies have documented cornulin expression in cSCC. In this study, we have established the patterns of cornulin expression in cSCC tissue samples that represent different histological grades of differentiation of cSCC. We hypothesize that an inverse relationship exists between cornulin expression and the degree of differentiation, as defined by the histopathological grading. Given that the lack of differentiation in tumors usually correlates to more aggressive behavior, our findings suggest that cornulin might serve as a diagnostic and prognostic indicator for cSCC.

## Materials and methods

Immunohistochemistry staining

Tissue microarrays were used in this study (US Biomax and US Biolabs, Rockville, MD) and contained de-identified tissue samples of normal skin and cSCC. The examiners were blinded to the histopathological grades of the cSCC tissue samples while performing the immunohistochemistry (IHC) staining and analysis.

IHC staining was performed on tissue microarray slides. Following antigen retrieval and blocking with 5% bovine serum albumin, the slides were stained with a 1:200 dilution of rabbit immunoglobulin (IgG) polyclonal primary antibody against cornulin (Sigma HPA024343; Sigma-Aldrich, St. Louis, MO), then labeled with 1:1,000 dilution of anti-rabbit horseradish peroxidase-conjugated secondary antibody (Sigma A9169; Sigma-Aldrich, St. Louis, MO). Finally, the tissue samples were incubated with 3,3’diaminobenzidine chromogenic substrate and counterstained with hematoxylin before being mounted with a coverslip.

Evaluation of cornulin expression

The IHC-stained tissue samples were imaged at high resolution using a Nikon A1R inverted microscope at 10× magnification. Computer-assisted image analysis was performed using Aperio ImageScope software (Leica Biosystems Inc., Buffalo Grove, IL) to avoid the observers’ subjectivity usually associated with the assessment of IHC staining intensity. A positive pixel counting v9 algorithm was utilized to quantify the immunoreactivity in the tissue region of interest. Regions of interest in each tissue sample were manually selected to include the normal epidermis or SCC cells and exclude the dermis or stroma, respectively. The algorithm then assigned red, orange, yellow, and blue colors to label strong (Nsp), medium (Np), weak (Nwp) positive pixels, and negative pixels, respectively. The number of Nwp, Np, and Nsp positive pixels; the total number of positive and negative pixels (NTotal); and the percentage of total positivity (NPositive/NTotal) were obtained. The percentage of weak, medium, and strong positivity was calculated by dividing Nwp, Np, and Nsp pixels by NTotal pixels, respectively. Immunoreactivity was determined based on the percentage of positively stained epithelial cells and staining intensity. Histo-score (H-score) was calculated by adding the percentage of positive cells multiplied by the weighted intensity of staining [18,19]. H-score = (1 × % weak positivity) + (2 × % medium positivity) + (3 × % strong positivity). The percentage of positive cells (% staining) value was obtained from the percentage of total positivity (NPositive/NTotal) value produced by the software. The average of H-scores for each compared group of samples was calculated, as well as the standard error of the mean (SEM), and compared using a two-sample unpaired t-test.

## Results

Cornulin expression in normal skin

**Figure 1:**
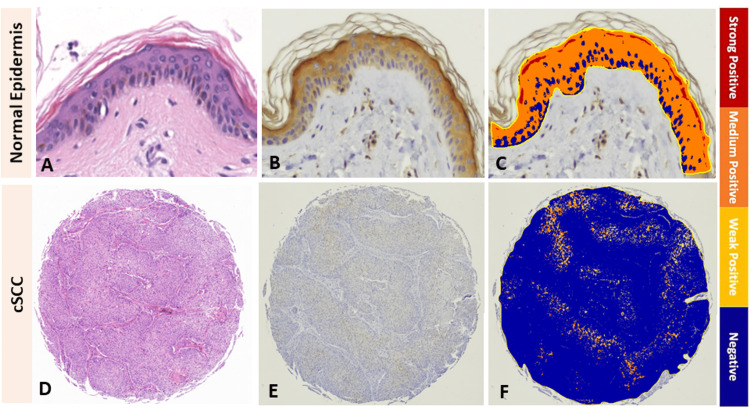
Cornulin expression in normal epidermis versus cutaneous squamous cell carcinoma tissue samples. Representative images of a normal epidermis: (A) H&E stained, (B) IHC for cornulin immunoreactivity visualized as cytoplasmic brown staining, (C) Image Scope analyzed, and representative images of a cutaneous squamous cell carcinoma tissue sample: (D) H&E stained, (E) IHC for cornulin immunoreactivity visualized as cytoplasmic brown staining, and (F) Image Scope analyzed. cSCC: cutaneous squamous cell carcinoma; H&E: hematoxylin and eosin; IHC: immunohistochemistry

IHC analysis showed measurable cornulin expression in the normal squamous epithelium of the epidermis with the strongest immunoreactivity in the upper epidermal layers (Figures 1A-1C). The mean H-score for cornulin immunoreactivity in the normal epidermis samples was 1.37.

Cornulin expression in cutaneous squamous cell carcinoma

**Figure 2:**
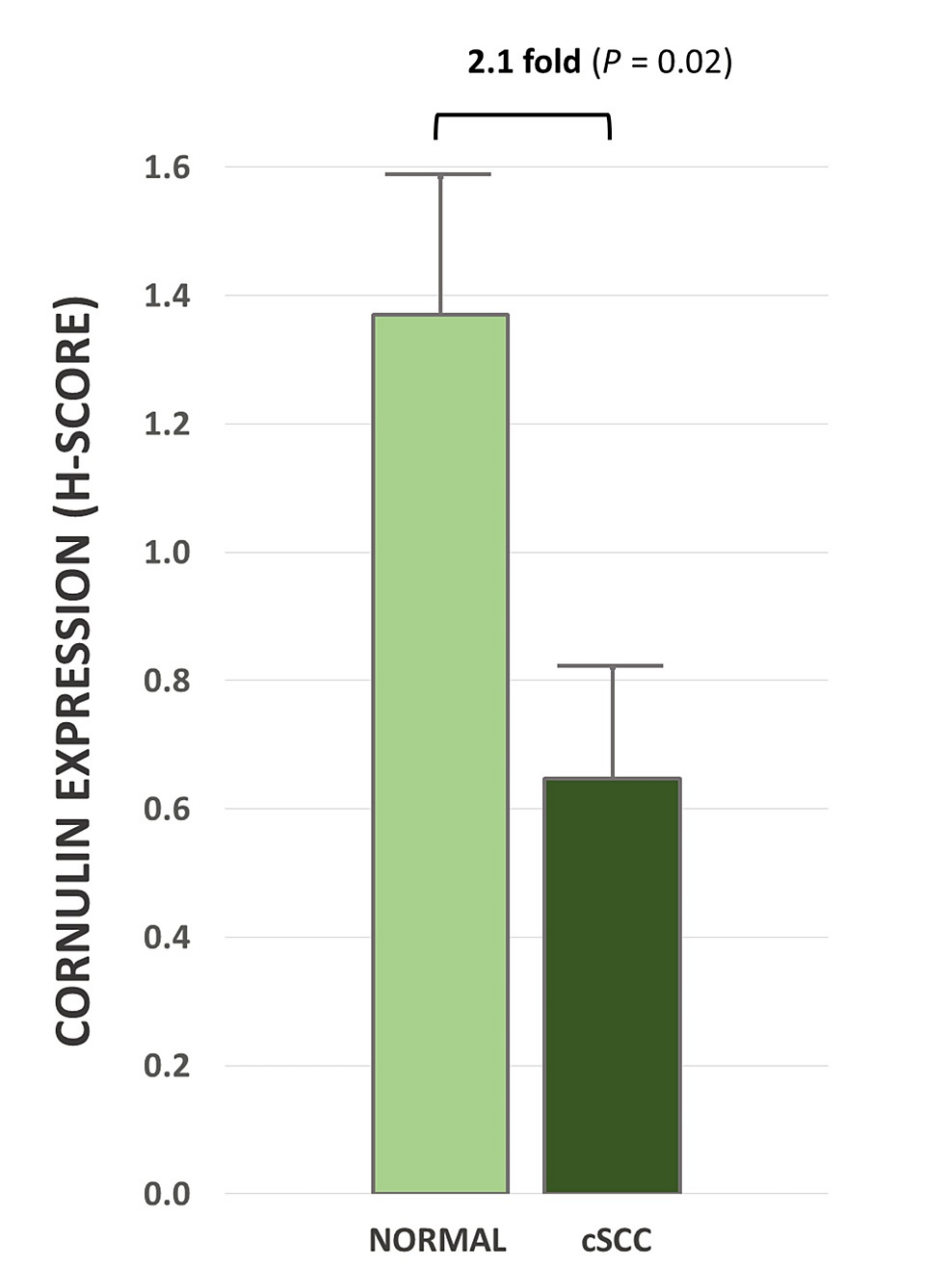
Cornulin expression as measured by mean H-scores in the normal epidermis versus cutaneous squamous cell carcinoma tissue samples. Bar graph depicting mean H-score for cornulin immunoreactivity in the normal epidermis samples (normal) (N = 7) and cSCC samples (N = 16). H-score: Histo-score; cSCC: cutaneous squamous cell carcinoma

In this study, we documented, for the first time, the expression of cornulin in cSCC (Figures 1D-1F). The mean H-score for cornulin immunoreactivity in cSCC tissue samples was 0.64, which represents a 2.1-fold downregulation compared to the normal skin samples (Figure 2). This overall downregulation of cornulin expression in cSCC was statistically significant (p = 0.02).

Correlation between cornulin expression and histopathological grade in cSCC

**Figure 3:**
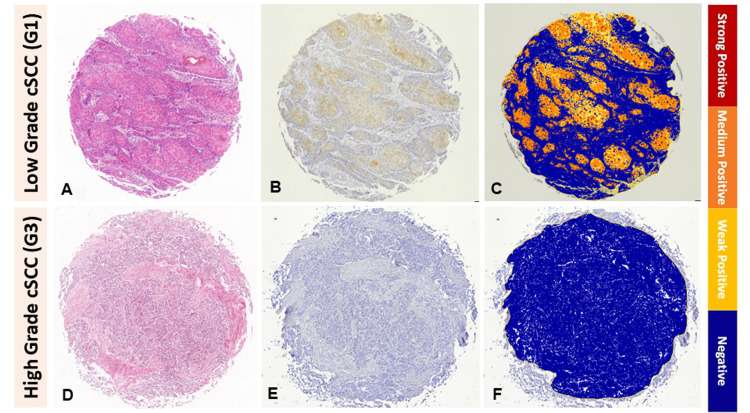
Cornulin expression in low histopathological grade cSCC versus high histopathological grade cSCC tissue samples. Representative low histopathological grade (G1) cSCC tissue sample: (A) H&E stained, (B) IHC for cornulin immunoreactivity visualized as cytoplasmic brown staining, (C) Image Scope analyzed, and representative high histopathological grade (G3) cSCC tissue sample: (D) H&E stained, (E) IHC for cornulin immunoreactivity visualized as cytoplasmic brown staining, and (F) Image Scope analyzed. cSCC: cutaneous squamous cell carcinoma; H&E: hematoxylin and eosin; IHC: immunohistochemistry

**Figure 4:**
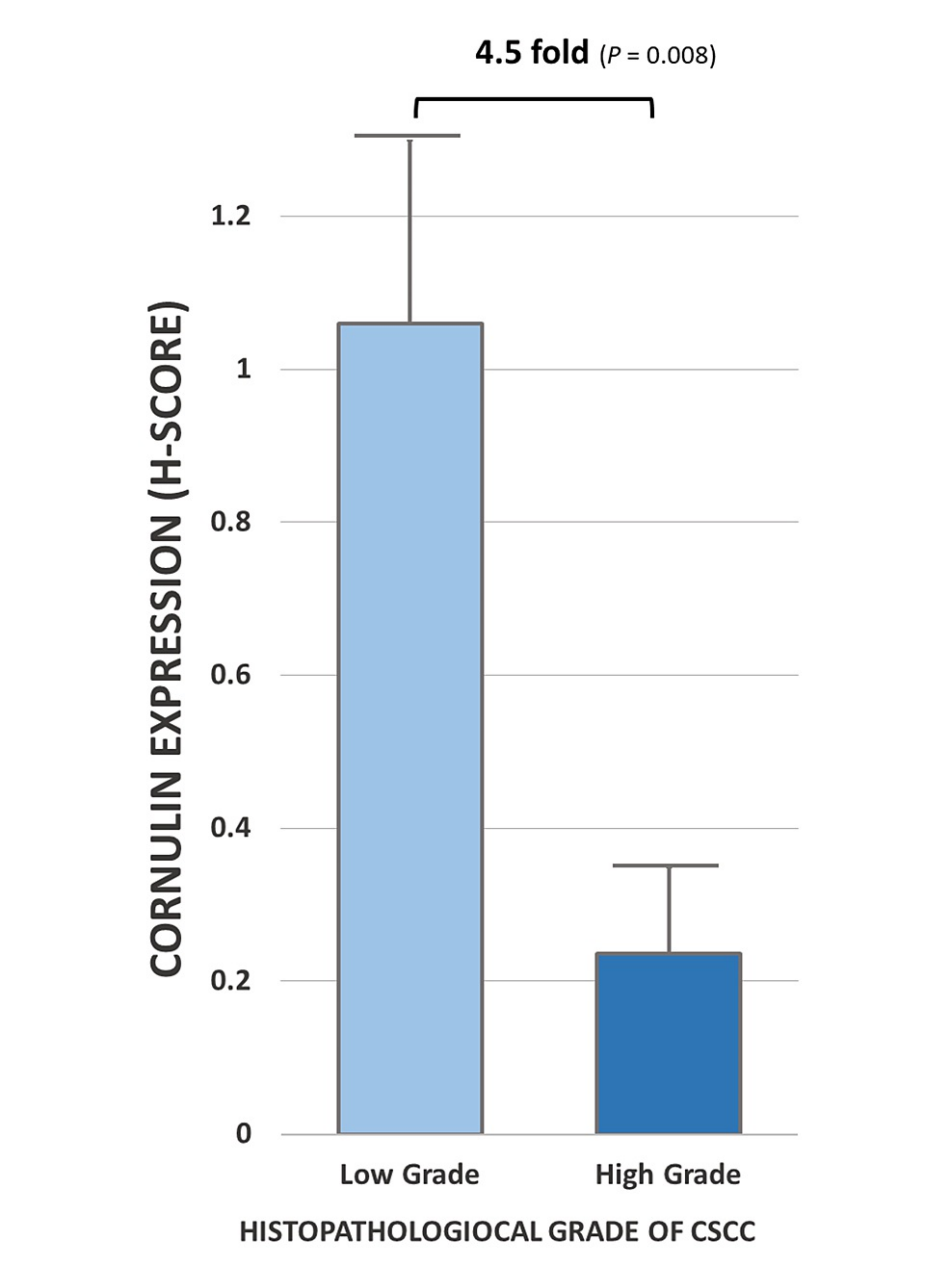
Cornulin expression as measured by mean H-scores in low histopathological grade versus high histopathological grade cSCC tissue samples. Bar graph depicting mean H-score for cornulin immunoreactivity in the low histopathological grade (G1) (N = 8) versus high histopathological grades (G2 and G3) (N = 8) tissue samples of cSCC. H-score: Histo-score; cSCC: cutaneous squamous cell carcinoma

**Figure 5:**
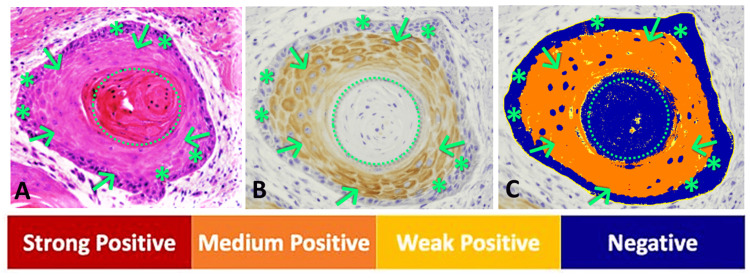
Cornulin expression around keratin pearls in well-differentiated cSCC tissue samples. Representative images of a keratin pearl (dotted circle) in a well-differentiated cSCC tissue sample; arrows represent keratinocytes adjacent to the keratin pearl; asterisks represent peripheral keratinocytes that are not directly adjacent to the keratin pearls: (A) H&E stained, (B) IHC for cornulin immunoreactivity visualized as cytoplasmic brown staining, and (C) Image Scope analyzed. cSCC: cutaneous squamous cell carcinoma; H&E: hematoxylin and eosin; IHC: immunohistochemistry

The mean H-score for cornulin immunoreactivity in well-differentiated cSCC tissue samples, designated as low histopathological grade G1, was 1.05, while the mean H-score was 0.23 for cornulin immunoreactivity in the combined moderately differentiated cSCC and poorly differentiated cSCC tissue samples, designated as high histopathological grades G2 and G3, respectively (Figure 3). Overall, tumors with high histopathological grades showed a statistically significant 4.5-fold decrease in cornulin expression, as measured by H-score, when compared to low histopathological grade tumors (p = 0.008) (Figure 4). Interestingly, the most intense cornulin staining was observed in malignant keratinocytes immediately adjacent to the keratin pearls as opposed to the peripheral keratinocytes that are not directly adjacent to the keratin pearls (Figure 5).

## Discussion

cSCC may not be as fatal as melanoma, but it is much more common, with its incidence rising in recent years [20]. Moreover, the metastasis rate is 37% for patients with high-risk features, such as poorly differentiated cSCC. Therefore, the accurate detection and characterization of this disease is paramount to successful treatment and enhanced survival rates [4]. Diagnostic methods for cSCC are heavily reliant on morphology-based histopathological examination. Currently, there are no established biomarkers to supplement the morphological examination of cSCC tumors. We conducted this study to determine the potential role of a novel biomarker, cornulin, as a diagnostic and prognostic indicator of cSCC. Using IHC and computer-assisted image analysis, we have shown that cornulin expression is downregulated by more than two-fold in cSCC compared to the normal epidermis.

Conventional histopathological grading of cSCC involves classifying samples into different categories based on morphological features such as keratinization, which indicates the tumor’s differentiation status. Grade I is considered well-differentiated and has >75% keratinization of the tumor. Grade II is moderately differentiated, which has 25-75% keratinization. Grade III is considered poorly differentiated with <25% keratinization. This grading system suffers from interobserver variability [21]. Thus, there is a need for quantitative biomarkers that can augment the predictive power in determining the grade of cSCC neoplasms. In this study, we investigated the correlation of cornulin expression with the differentiation status of cSCC. Our findings show that cornulin was downregulated by 4.5-fold in tumors with high histopathological grades when compared to low histopathological grade tumors. Therefore, we postulate that cornulin expression can be utilized as an adjunct to pathological examination to evaluate the differentiation status of cSCC specimens. Gene expression profiles designed to help stratify cSCC risk are growing in popularity, and we believe that cornulin expression could be a beneficial addition to these profiles in detecting and staging cSCC [22].

## Conclusions

In this study, we have documented the expression of cornulin in cSCC for the first time. Cornulin proved to be significantly downregulated when compared to normal skin. Moreover, cornulin expression correlated significantly with the differentiation status of cSCC. We have shown that cornulin expression levels significantly decline as differentiation is lost in tumors with high histopathological grades. Altogether, the measurement of cornulin expression aided in distinguishing between the low histopathological grade and the high histopathological grades, making it a potential adjunct tool for grading cSCC lesions. Longitudinal studies are needed to establish the utility of cornulin as a diagnostic and prognostic biomarker by testing in large-scale tissue sample sets that are associated with medical history and clinical follow-up data.
